# Oral mucosal peeling related to dentifrices and mouthwashes: A systematic review

**DOI:** 10.4317/medoral.22939

**Published:** 2019-06-28

**Authors:** Daniel Pérez-López, Pablo Varela-Centelles, María J. García-Pola, Pablo Castelo-Baz, Lucía García-Caballero, Juan M. Seoane-Romero

**Affiliations:** 1 Dept of Surgery & Medical-Surgical Specialities. School of Medicine & Dentristry. University of Santiago de Compostela; 2 CS Praza do Ferrol. EOXI Lugo, Cervo, e Monforte. Servizo Galego de Saúde. Lugo. Spain; 3 Dept of Surgery & Medical-Surgical Specialities. School of Medicine & Health Sciences. University of Oviedo. Spain

## Abstract

**Background:**

The aim of this systematic review was to summarise the clinical information available about oral mucosal peeling (OMP) and to explore its aetiopathogenic association with dentifrices and mouthwashes.

**Material and Methods:**

PICOS outline: Population: subjects diagnosed clinically and/or pathologically. Intervention: exposition to oral hygiene products. Comparisons: patients using products at different concentrations. Outcomes: clinicopathological outcomes (primary) and oral epithelial desquamation (secondary) after use. Study design: any. Exclusion criteria: reports on secondary or unpublished data, in vitro studies. Data were independently extracted by two reviewers.

**Results:**

Fifteen reports were selected from 410 identified. Descriptive studies mainly showed low bias risk, experimental studies mostly an “unclear risk”. Dentifrices or mouthwashes were linked to OMP, with an unknown origin in 5 subjects. Sodium lauryl-sulphate (SLS) was behind this disorder in 21 subjects, tartar-control dentifrices in 2, and flavouring agents in 1 case. Desquamation extension was linked to SLS concentration. Most cases were painless, leaving normal mucosa after desquamation. Tartar-control dentifrices caused ulcerations more frequently.

**Conclusions:**

OMP management should consider differential diagnosis with oral desquamative lesions, particularly desquamative gingivitis, with a guided clinical interview together with pathological confirmation while discouraging the use of the product responsible for OMP.

** Key words:**Systematic review, oral mucosal peeling, dentifrices, sodium lauryl-sulphate, oral hygiene products.

## Introduction

A possible relationship between oral epithelium desquamation and dentifrices was hypothesized in early 1970s by Pnacek (1), and very few cases have been reported ever since (2-4), although oral mucosal peeling (OMP) induced by oral hygiene products seems to be a relatively common finding in clinical practice (5).

Several agents have been linked to adverse oral mucosal reactions, but attention has focused on detergents, particularly sodium lauryl sulphate (SLS) (3,6-10), which has been reported to induce OMP together with mucosal inflammation, higher permeability to chemicals, and denaturation of proteins (11). Neither the aetiopathogenesis of OMP nor its clinicopathological features are well established (11-15). It is often unrecognised because its asymptomatic nature results in some patients deciding to ignore it while others are distressed because clinicians are sometimes unable to identify a cause or to establish treatment (11,16). However, -and considering differential diagnoses of OMP include erosive oral disorders such as lichen planus, autoimmune alterations, candidosis, mechanical, thermal or chemical trauma, adverse drug reactions, viral infections, and recurrent aphtous ulcerations- establishing a definitive diagnosis is paramount (11,17).

In this situation, where the aetiopathogenesis is poorly understood, the clinical presentation is often unrecognised, the diagnosis and treatment are not soundly founded, along with the lack of previous reviews on this topic justify the need for a systematic review on the relationship of OMP with the use of dentifrices and mouthwashes. Thus, the aim of this systematic review was to summarise the clinical information available about oral mucosal peeling and to explore its aetiopathogenic association with dentifrices and mouthwashes.

## Material and Methods

The review protocol was agreed by all authors and registered with the International Prospective Register of Systematic Reviews (PROSPERO, No: CRD42018103792) (18). This systematic review was performed according to the PRISMA (Preferred Reporting Items for Systematic Reviews and Meta-analysis) guidelines (19) and followed the outlines of PICOS (20): i) Population: subjects clinically and/or pathologically diagnosed of OMP; ii) Intervention: exposition to oral hygiene products; iii) Comparisons: patients using different dentifrices and mouthwashes at different concentrations under experimental conditions; iv) Outcomes: primary outcome: clinical and histological outcomes after using dentifrices and mouthwashes. Secondary outcome: desquamation of oral epithelium after exposition to oral hygiene products; v) Study design: any type.

-Information sources and systematic search

Medline (PubMed) and Embase (Ovid) were searched on 19 June 2018, together with ISI proceedings for both English and non-English written articles. The search was supplemented by scanning the reference lists of relevant review papers identified as well as those of the articles of finally included in this systematic review. We also explored the authors’ personal files to make sure that all relevant material had been captured. Three authors were contacted for further information and all of them attended our request (2,3,21).

The search was updated on 8 November 2018. Search strategy: ((“mouth mucosa”[MeSH Terms] OR (“mouth”[All Fields] AND “mucosa”[All Fields]) OR “mouth mucosa”[All Fields] OR (“oral”[All Fields] AND “mucosa”[All Fields]) OR “oral mucosa”[All Fields]) AND (peeling[All Fields] OR shedding[All Fields] OR epitheliolysis[All Fields] OR desquamation[All Fields])) OR (((“toothpastes”[MeSH Terms] OR “toothpastes”[All Fields] OR “toothpaste”[All Fields]) OR (“dentifrices”[Pharmacological Action] OR “dentifrices”[MeSH Terms] OR “dentifrices”[All Fields]) OR (“mouthwashes”[Pharmacological Action] OR “mouthwashes”[MeSH Terms] OR “mouthwashes”[All Fields])) AND ((“adverse effects”[Subheading] OR (“adverse”[All Fields] AND “effects”[All Fields]) OR “adverse effects”[All Fields] OR (“side”[All Fields] AND “effects”[All Fields]) OR “side effects”[All Fields]) OR (“adverse effects”[Subheading] OR (“adverse”[All Fields] AND “effects”[All Fields]) OR “adverse effects”[All Fields])) AND (“mouth mucosa”[MeSH Terms] OR (“mouth”[All Fields] AND “mucosa”[All Fields]) OR “mouth mucosa”[All Fields] OR (“oral”[All Fields] AND “mucosa”[All Fields]) OR “oral mucosa”[All Fields]))

-Eligibility criteria

Inclusion: clinical and experimental reports addressing OMP induced by dentifrices and mouthwashes. No limitations by language or publication date were introduced. Exclusion: papers reporting on secondary data, in vitro studies, and unpublished data.

-Data collection and extraction

Data extraction was independently performed by two reviewers (DPL & PVC) and results summarized in standardized forms for descriptive and experimental studies. Inter-observer concordance was calculated using the Epidat 3.1 statistical package (Programa para Análisis Epidemiológico de Datos Tabulados. Xunta de Galicia. Santiago de Compostela. Spain). Reports identified through the searches were exported to Refworks (ProQuest, Santiago de Compostela, Spain) and checked for duplicates. Papers were filtered by title and the relevant ones retrieved for further analysis. The reasons for exclusions were recorded at each step.

Data retrieved for descriptive studies included: authors and year of publication, study type, number of participants, age, gender, medical and dental history, medication, oral hygiene products, symptomatology, site of the lesion, evolution time, diagnostic methods, histopathological findings, and management. In the case of experimental studies, the following information was recorded: authors and year of publication, study type, number of participants, inclusion / exclusion criteria, age, gender, exposition variable / oral hygiene products, result variable / adverse effects, site of the lesion, study periods, and diagnostic methods.

-Quality assessment

The Cochrane Collaboration´s tool for assessing risk of bias was used for experimental studies (22), which includes 7 dominions: Random sequence generation (selection bias), allocation concealment (selection bias), blinding of participants and personnel (performance bias), blinding of outcome assessment (detection bias), incomplete outcome data (attrition bias), selective reporting (reporting bias), and other sources of bias. Case reports and case series was evaluated by means of the instrument proposed by Murad et al (23), which considers 4 specific dimensions: selection, ascertainment, causality, and reporting. The risk for bias was grouped under the following categories: Low risk: low risk of bias for all domains; Unclear risk: unclear risk for one or more domains; and High risk: high risk for one or more domains. Quality was independently assessed by two reviewers (DP & PV), who solved disagreements by discussion until a consensus was reached.

## Results

-Study selection

**Figure 1:**
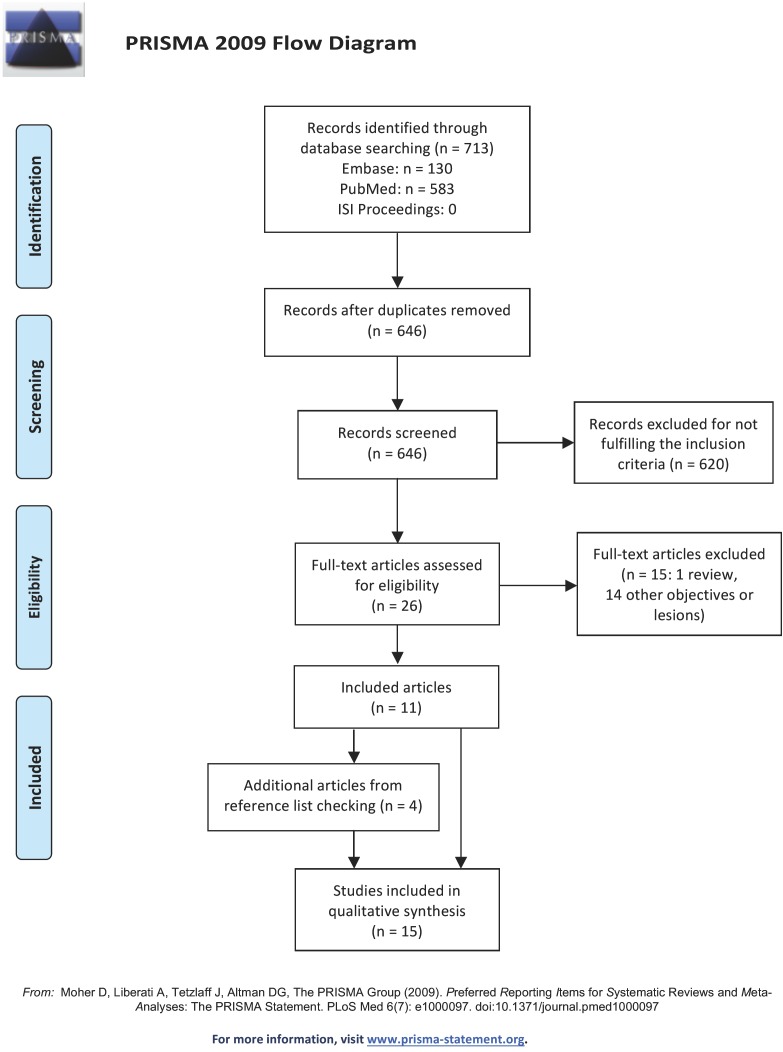
PRISMA flow diagram.

We identified 713 potentially eligible reports, and 646 remained after adjusting for duplicates. Another 341 of them were discarded after checking their abstracts (kappa= 0.659). The full texts of the remaining 20 reports were retrieved and examined. As a result, 11 reports were selected (Kappa= 0.813). Four additional papers were identified by screening the reference lists of these reports, so a total of 15 articles were finally included in this systematic review (Fig. 1).

-Risk of bias in included studies

Descriptive studies were mainly found to be at a low risk (2,3,6,11,24,25), as only three papers scored a high risk for bias (4,5,16). Regarding experimental studies, five reports were classified as “unclear risk for bias” (7,21,26,27), and the other two selected papers were found to be at high (28) and low (29) risk respectively.

-Descriptive studies

Six case reports (5,6,16,11,24,25) and 3 case series (2-4) were selected ( Table 1), all published in English but one, in German. Most papers were authored in the States -6 papers (4-6,11,24,25)-. The remaining reports came from Germany (3), Jordan/United Kingdom (16), and Italy (2).

**Table 1:**
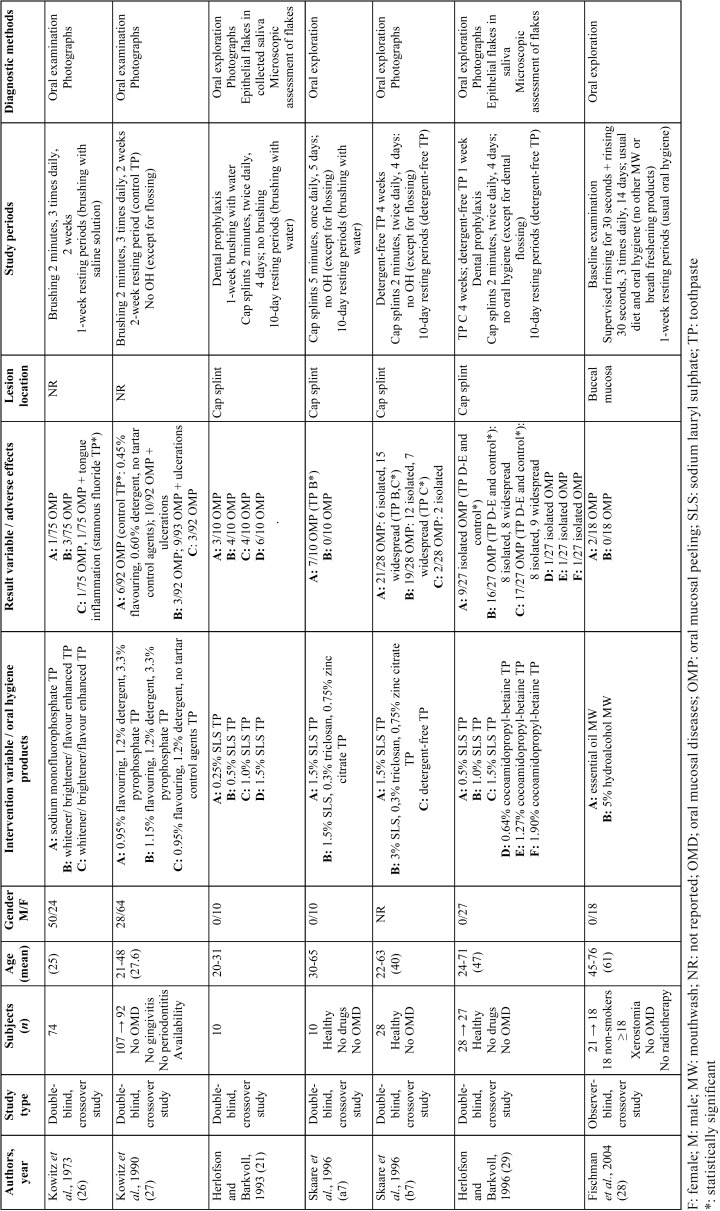
Data extraction for experimental studies.

The selected descriptive studies reported on 28 subjects, mostly -20- females. Age ranges varied from 47-72 years for 6 subjects (3), to 27-80 (2,4-6,16,24,25), with an average of 49.5.

Systemic conditions were reported for 6 individuals who were under medication, although these drugs were rather heterogeneous and not consistent among case reports (4,6,25,26). Dentifrices or mouthwashes were found to be related to this phenomenon in 23 individuals (2,3,5,6,24,25), while the causal agent could not be identified in 5 subjects (4,16). Histopathological characteristics (3,4,6,24,25) and management strategies (2-6,16,24,25) were described in 25 cases.

Most subjects were affected by painless OMP showing otherwise normal mucosa after desquamation (23/28). In addition, 2 case reports described the same symptomatology without providing data about the remaining tissue after desquamation (5,24). Painful erosions after peeling were present in the remaining 3 subjects (3,6), who reported burning or dry mouth sensation in two (5,24) and one (25) individuals, respectively.

OMP was linked to SLS toothpastes in 21 subjects (2,3,6,24,25), while tartar control dentifrices were associated to another 2 cases (5,24), where flavouring agents were held responsible in one of them (24).

Lesions were most frequently located in the alveolar mucosa, vestibules and buccal mucosa (2,4,6,16,24,25). Seven patients showed widespread lesions throughout the oral cavity (3).

The time of OMP evolution varied from 3 days to 10 years. This information was missing for 3 subjects (4-6).

Incisional and peeling biopsies were performed in 7 (3,4,6,24,25) and 19 (2-4) subjects, respectively. The former usually revealed a superficial intraepithelial linear cleft accompanied by parakeratosis, mild acanthosis, intracellular oedema, normal connective tissue, and negative direct immunofluorescence. Peeling biopsies showed an epithelium composed of 2-15 layers with thin elongated strips of parakeratotic material and some polyhedral squamous cells with lightly stained cytoplasms; nor dysplasia nor vesicles were present.

Fungal staining was performed in 6 incisional (3,4,24,25) and 7 peeling biopsies (3). All were negative but one surgical specimen that showed PAS-positive material within the epithelium, but no yeast forms were seen (25).

The main treatment of OMP induced by oral hygiene products was discontinuation of the causal toothpaste or mouthwash (2,3,5,6,24,25), sometimes accompanied by the use of a regular (5,24) or biologically inert dentifrice (25). Resolution time ranged between 24 hours and 3 weeks (2,3,6).

-Experimental studies

Six articles involving 7 experimental studies were finally selected for the review (7,21,26-29). All were published in English: three papers came from Norway (7,21,29) and 3 from the USA (26-28) ( Table 2).

**Table 2:**
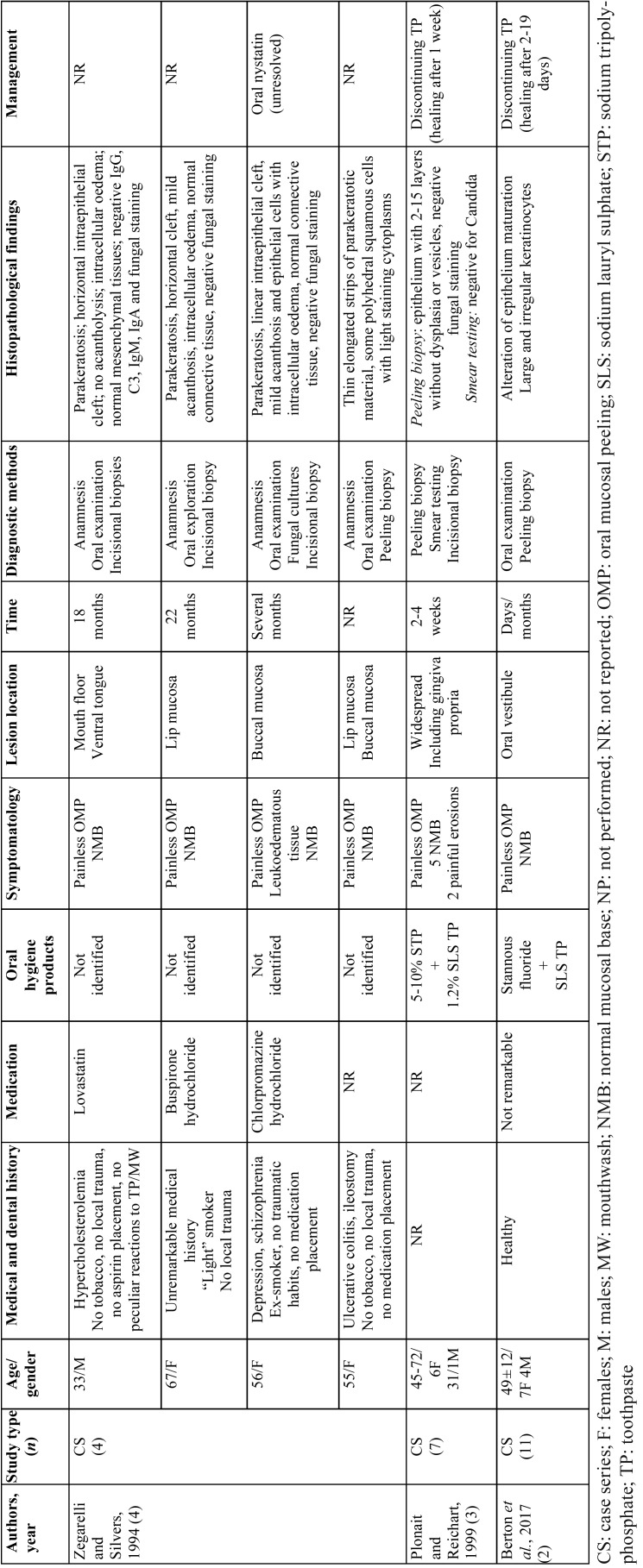
Data extraction for descriptive studies.

Toothpaste studies mainly consisted of two methodologies: three studies applied a toothpaste-containing cap splinted to the upper jaw during 2 minutes, twice a day for 4 days (7,21,29), while another study employed the same method for 5 minutes, once a day for 5 days (7) In addition, 2 more studies asked subjects to brush their teeth for 2 minutes, 3 times per day for 2 weeks (26,27). The mouthwash study design consisted of a supervised 30-second rinsing plus homemade rinses for the same time, 3 times a day for 2 weeks (28).

Most studies focused on dentifrice adverse effects on oral mucosa, mainly desquamation -6 out of 7- (7,21,26,27,31), while mouthwashes were investigated in a single report (28). The most frequently examined ingredient was 0.25-3 % SLS -4 out of 6- (7,21,29). Essential oils were analysed in the mouthwash study (28).

Four studies have demonstrated a causal relationship between SLS-containing toothpastes and OMP, even at low concentrations (7,21,29). SLS in concentrations of 0.5 %, 1.0 %, and 1.5 % were responsible for 13/37, 20/37 and 51/75 oral mucosal desquamations, respectively (7,21,29).

Differences in the occurrence of OMP between 1.5 % SLS and detergent-free toothpastes resulted significant in one study (7), while other investigation reported this difference even with 0.5 % SLS dentifrices (29).

Additionally, SLS toothpastes have been significantly related to higher incidence of OMP when compared to the alternative detergent cocoamidopropyl-betaine (29).

Furthermore, the extent of desquamation has been significantly linked to SLS concentration. In this vein, two studies grading the severity of OMP have shown toothpastes with 1.5 % SLS were responsible for 14/55 isolated and 24/55 widespread desquamative reactions (7,29). The addition of triclosan in concentrations of 0.3 % has been demonstrated to significantly reduce the number of desquamative reactions compared to 1.5 % SLS as well as their severity with 3 % SLS (7).

On the other hand, a stannous fluoride dentifrice has been reported to produce a significant lower level of oral adverse reactions (desquamation, ulceration, inflammation and erythema) than whitener/brightener/flavour-enhanced, sodium fluoride toothpastes (26).

Tartar control toothpastes have been associated with a higher number of ulcerations, sloughing, and erythema than dentifrices including the same concentrations of flavourings and detergents but without pyrophosphate (27). This latter report focused on desquamation found a significant difference only between a dentifrice containing 3.3 % pyrophosphate and a toothpaste without tartar control agents (27).

## Discussion

The limitations inherent to the reduced number of patients reported in uncontrolled and retrospective case reports/case series requires a cautious interpretation of their data. However, experimental studies, mainly at low or unclear risk of bias, provided information robust enough to state OMP induced by oral hygiene products is mainly linked to SLS-containing dentifrices, followed by tartar-control formulations (3,5-7,21,24-27,29).

It is worth mentioning the wide variety of terms used to designate this clinical condition: desquamative stomatitis (6), oral mucosal desquamation (2,21,29), oral slough (25), and shedding oral mucosa (4), but the term “oral mucosal peeling” (OMP) is the one which gathers the largest proportion of cases and has gained acceptance within the scientific community (3,16).

-Aetiopathogenesis

It has been stated that higher portions of anionic detergents are likely to break up the intercellular structure of the epithelium causing increased epithelial cell desquamation, thus suggesting a toxic reaction in the genesis of OMP (6,10). In fact, the peeling phenomenon has found to be analogous to that in certain thermal of chemical injuries, such as aspirin burn, but in a milder form (4,24). Our results show SLS, even at concentrations as low as 0.25%, is related to the occurrence of desquamation with a dose-response effect. Moreover, the differences in irritative potential between SLS and other detergents may be partially explained by the stronger protein denaturation effect of anionic detergents (15).

Different critical micellar concentrations, adsorptive properties, and effects on the biomembrane system have been also described as responsible for these variations (12-14), together with an influence of pH (3,6).

Undiluted SLS-containing toothpastes can be retained in the oral cavity after brushing with biological effects of SLS for at least 2 hours after toothbrushing (30), but with only minimal adverse oral mucosal reactions (31).

Interestingly, triclosan has been demonstrated to reduce the number of desquamations and their severity by reducing the penetration of SLS molecules through the mucosa, stabilizing and protecting the superficial epithelial cells from irritation and inhibiting the cascade of inflammatory reactions (7,32). Zinc may also have a stabilizing effect on the mucosa (33). Additionally, alterations in oral mucosal permeability and absorption with age may be explained by differences in mucosal response to SLS in pre- and post-menopausal women (34).

Apart from that, the concomitant effect of other ingredients, such as stannous fluoride or sodium tripolyphosphate, require further investigation (2,35). The part of tartar control agents in OMP and the possible role of flavourings also needs to be elucidated (24,27).

-Clinical appearance

**Figure 2:**
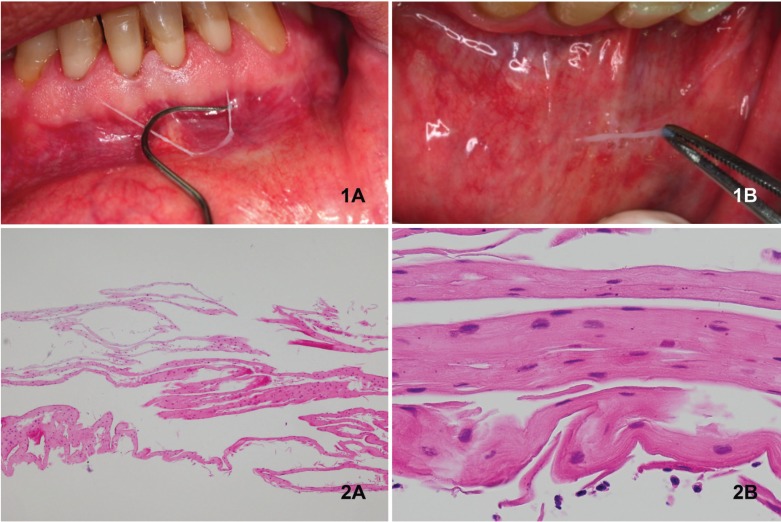
1A: Epithelial desquamation in alveolar mucosa; 1B: Peeling biopsy technique in the inner side of lower lip: 2A: Parallel sheets of flattened surface layers corresponding to normal gingival stratified squamous epithelium (HE x4); 2B: At greater magnification, cells exhibited good maturation with round/elliptical to flattened nuclei of the superficial cells (HE x40).

OMP induced by oral hygiene products usually displays a grey-whitish strips of oral epithelium that either slough spontaneously or can be easily peeled off exposing an otherwise normal tissue (3-5,16,24,25) (Fig. 2). Occasionally, this mucosa shows a whitish colour similar to leukoedema (4,25). This condition is frequently asymptomatic but painful erosions after peeling may also be present (3,6).

There is no gender or age pattern, although it is slightly more frequent among females in their 50s. No specific medical or dental conditions could be related to OMP (2-6,16,24,25). Although various oral sites are usually affected, most frequently involved areas include oral vestibule, buccal mucosa and gingiva. The evolution time is quite variable, from 3 days to 10 years, depending on the exposition to the causal agent (2-6,16,24,25).

-Histopathological features

Studies relying on incisional biopsies have shown a constant pathological pattern for OMP characterized by a parakeratotic epithelium, mild acanthosis and intracellular oedema. A linear, horizontal intraepithelial cleft without acantholysis can also be observed together with absent or minimal inflammation of connective tissue. Negative direct immunofluorescence and fungal positive staining can usually be observed (3,4,6,24,25).

-OMP as desquamative gingivitis

Gingival desquamation, as a single location or associated to desquamation of alveolar mucosa, lateral, ventral or dorsal tongue, inner lip mucosa, floor of the mouth, vestibules and buccal mucosa, has been widely described in the realm of OMP (3,5,6,25). Thus, OMD complies with the criteria for desquamative gingivitis (DG) but this entity neither is a specific disorder nor has a single aetiopathogenia. DG represents a wide group of mucocutaneous and systemic pathologies with gingival involvement (35-38), mainly mucous membrane pemphigoid (MMP), oral lichen planus, and pemphigus vulgaris (35-38), but including also erythema multiforme, graft-versus-host disease, lupus erythematosus, chronic ulcerative stomatitis, plasma cell gingivitis, linear IgA disease, dermatitis herpetiformis, epidermolysis bullosa, paraneoplastic forms, psoriasis, and foreign body gingivitis, together with drug induced forms (37,38) in minor frequencies. A reasonable addition to this already large list of disorders grouped under the heading of DG would be OMP.

A definitive diagnosis of DG requires a thorough clinical examination including specific blood tests and, frequently, pathologic and immunopathologic assessments.

In this vein and considering that clinical signs and symptoms are insufficient to establish a definitive diagnosis, the stab-and-roll biopsy technique would make an important contribution to pathological diagnosis preserving gingival epithelium in the specimen, which is crucial for an accurate DG diagnosis (35).

However, considering peeling biopsy technique can harvest enough epithelium for both pathological and immunofluorescence studies, it could be a sound alternative -with less morbidity- to reach a definitive diagnosis of the cause behind DG. In non-conclusive cases, an incisional biopsy should be undertaken (36).

-Diagnosis and management of OMP

Diagnosis of OMP is based upon a comprehensive intraoral exploration and also on a clinical interview focused at identifying topical substances -including oral hygiene products- with a potential to induce desquamative oral lesions (37,38). In addition, and once a clinical judgement was established, an “ex juvantibus” approach could also be attempted by discontinuing the use of the potentially involved products.

Peeling and incisional biopsies, as well as smear testing, are the most frequently used diagnosis methods (2-6,16,24,25). Incisional biopsies have been also extensively used to reach a definitive diagnosis of OMP (3,4,6,24,25), although similar certainty in diagnosis can be achieved by peeling biopsy (3,4) (Fig. 2).

OMP does not require any kind of treatment beyond the discontinuation of the responsible toothpaste or mouthwash when the peeling phenomenon causes discomfort (2,5,6,16,24,25).

Clinical practitioners are in an advantageous position in the prescription of oral hygiene products and, therefore, in the diagnosis of this disorder. Awareness of OMP permits a rapid and costless differential diagnosis with other DGs. Besides, the use of a peeling biopsy technique ensures diagnosis in a more respectful way to the periodontium than alternative, incisional biopsy techniques.

## Conclusions

With the limitations inherent to the type of studies included in this systematic review, it can be concluded that there is a causal relationship between toothpastes and mouthwashes with oral mucosal peeling, mostly due to high concentrations of sodium lauryl sulphate in their formulations, although other components of oral hygiene products can also be held responsible for this disorder in much lower frequencies.

Clinical management of OMP should consider differential diagnosis with other oral desquamative lesions, particularly DG, and include a guided clinical interview together with pathological confirmation through peeling biopsy along with discouraging the use of the product responsible for OMP.
